# Depressive symptoms among older adults with diabetes mellitus: a cross-sectional study

**DOI:** 10.1590/1516-3180.2021.0771.R5.09082022

**Published:** 2022-10-03

**Authors:** Diego Micael Barreto Andrade, Roseanne Montargil Rocha, Ícaro José Santos Ribeiro

**Affiliations:** I PhD. Nurse and Professor, Faculty of Health Sciences, University of Pécs (UP), Pécs, Hungary.; II PhD. Nurse and Full Professor, Department of Health II, Universidade Estadual de Santa Cruz (UESC), Ilhéus (BA), Brazil.; III PhD. Nurse Researcher and Professor, Department of Health II, Universidade Estadual do Sudoeste da Bahia (UESB), Jequié (BA), Brazil.

**Keywords:** Depression, Primary health care, Osteoporosis, Diabetes mellitus, Diabetes complications, Depressive symptoms, Diabetic complications, Geriatric depression

## Abstract

**BACKGROUND:**

Diabetes mellitus is a chronic disease with long-term consequences that is often associated with depressive symptoms. This relationship predicts increased morbidity and mortality rates, leading to serious health consequences.

**OBJECTIVE:**

To identify the prevalence and health factors associated with depressive symptoms among older adults with diabetes mellitus.

**DESIGN AND SETTING:**

An observational cross-sectional study was conducted among 236 older adults in the Basic Healthcare Units of Jequié, Brazil.

**METHODS:**

A survey containing sociodemographic, behavioral, and health conditions was used as a data collection instrument, in addition to the Geriatric Depression Scale. The main inclusion criterion was older adults diagnosed with diabetes mellitus. To identify the risk factors associated with depressive symptoms among older adults with diabetes mellitus, logistic regression analysis was conducted for calculating the odds ratio (OR), and a 95% confidence interval (CI) was considered statistically significant.

**RESULTS:**

The prevalence of depressive symptoms was 24.2% among older adults with diabetes, corroborating the Brazilian average of 30%. The final multivariate analysis model for the risk of depressive symptoms showed a significant association with diabetes complications [OR = 2.50, 95% CI 1.318–4.74)] and osteoporosis [OR = 2.75, 95% CI 1.285–5.891)].

**CONCLUSION:**

A high prevalence of depressive symptoms was observed among older adults with diabetes. Critically examining older adults with diabetes mellitus is necessary, and screening for depressive symptoms is highly recommended, especially for those with complications resulting from diabetes mellitus and musculoskeletal comorbidities, such as osteoporosis, as it seems to be associated with depressive symptoms.

## INTRODUCTION

Diabetes mellitus (DM) is a chronic disease that primarily affects older adults. Owing to long-term consequences, such as complications of the kidneys, eyes, nerves, heart, and blood vessels, DM constitutes a major public health problem.^1,2^ The prevalence of diabetes is increasing worldwide. According to the International Diabetes Federation's 2021 Diabetes Atlas, 537 million adults aged between 20 and 79 years are living with diabetes. In Brazil, estimates show that up to 16.8 million people have DM, which is approximately 7% of the population.^1^

Moreover, the presence of depressive symptoms deserves equal attention because of its increasing prevalence among community-dwelling older adults, ranging from 13% to 39%.^3^ The prevalence of depressive symptoms in Jequié, Bahia, Brazil, exceeded 88% of older adults, and was mostly correlated with chronic diseases.^4^ Conversely, there are high rates of depression underdiagnosis in older adults, which can increase the development of other risk factors in this population.^5–7^

Several studies have suggested an association between diabetes and depression. There are various predictors of depression among older adults with DM, such as socioeconomic, individual, behavioral, and clinical factors.^8^ Depression has been reported as a risk factor for type 2 diabetes.^9,10^ Meanwhile, depression is reportedly two times more prevalent in people with DM than in people who do not have diabetes.^11–13^ Depression has also been linked to family dysfunction and poor health outcomes in patients with type 2 diabetes.^12,14–16^

Nonetheless, depression and diabetes represent the fourth and eighth most important causes of disability-adjusted life years, respectively.^17^ Moreover, this relationship predicts increased morbidity and mortality rates, non-adherence to treatment, low quality of life, and an immense public health impact.^11,12,18–20^

Therefore, this study is important because, globally, depressive symptoms and diabetes in older adults are becoming the leading causes of disability, with greater frailty and vulnerability. Thus, the presence of depressive symptoms associated with DM can seriously impact an individual's physical health and quality of life, since both increase their risk for mortality and poor disease management. Furthermore, primary care is the gateway to identifying and monitoring individuals with DM. Thus, this study is relevant to help identify risk factors, establish early interventions, and plan appropriate care for these individuals. Our research questions were: “What is the prevalence of depressive symptoms among older adults with DM?” and “What is the relationship between depressive symptoms and health conditions in older adults?” We hypothesized that a significant proportion of depressive symptoms among older adults with DM would be related to their health status.

## OBJECTIVE

This study aimed to identify the prevalence of and health factors associated with depressive symptoms in older adults with DM.

## METHODS

### Study design and setting

This cross-sectional study was conducted among 236 older adults enrolled and registered in the Monitoring and Control Service of Hypertension and Diabetes at four Basic Healthcare Units (BHU) in the city of Jequié, in the southwest region of the State of Bahia, Brazil. The estimated population of Jequié is 156,277, with approximately 17,000 older adults aged 60 years or older. Among them, more than 10,000 were assisted under the BHU, and the remaining older adults were distributed between family health strategy units and private healthcare.^21^

### Sample

To compose the sample, the E-SUS Component Individual Care Form was used to group individuals with diabetes aged 60 years or older. This is an online registration form that contains patients' personal information regarding their health problems/conditions and is acquired during individual consultations with primary care professionals. After grouping, a sample of 813 individuals was identified. Adopting a 95% confidence level, 5% error, factor prevalence (i.e., depressive symptomatology) of 30.0 %,^22^ and 20% loss replacement rate, a sample of 236 individuals was calculated.

The research was conducted in four BHU areas, containing a total of 91 micro-areas. We conducted a simple random draw from the micro-areas, and the respective community health agent was recruited to help during the home visits and assist the research team in locating the residences. In case of the unavailability or absence of older adults with diabetes in the micro-area, the next micro-area was selected, following the survey for older adults with diabetes until saturation was reached for the number of individuals by BHU.

Inclusion criteria were older adults with DM type 2, aged 60 years or older, and who were enrolled in the BHU area and registered in the Monitoring and Control Service of Hypertension and Diabetes. Exclusion criteria were older adults with cognitive difficulties as established by the Mini-Mental State Examination.

### Data collection

For data collection, a form comprising two survey sets was applied, including sociodemographic, behavioral, and health conditions, along with the Geriatric Depression Scale (GDS-15).

#### Dependent variable

For analysis, depressive symptoms were used as the dependent variable. The Brazilian version of the GDS, abbreviated to 15 items, was used in this study. Regarding the definition of depressive symptoms, scores of ≤ 5 points = negative (absence of depressive symptoms) and ≥ 6 points = positive (presence of depressive symptoms).^23^

#### Independent variables

The sociodemographic variables collected were sex (male and female); age in years tabulated in age groups (60–69, 70–79, and 80 years or older); ethnicity (white, brown, black, and others); marital status (with partner, without partner); and education level divided into two groups (elementary school and above, primary school and below).

The behavioral variables collected were physical activity (yes or no); smoking habits (never smoked, former smoker, and smoker); alcohol habits (non, moderate, excessive consumer); practicing any religion (Catholic, Protestant, and not practicing); and financial difficulty (yes or no).

The health conditions were assessed dichotomously (yes or no), pertaining to family history of diabetes; diabetes complications; rheumatism; osteoporosis; systemic hypertension; circulation problems; heart problems; difficulty sleeping; vision problems; chronic pain; type of DM complications (renal, ocular, circulatory, diabetic foot, and amputation); and prescribed treatment (oral, insulin, non-medicated, none).

### Data analysis

Descriptive analysis of population characteristics was performed for all continuous variables (described as mean and standard deviation values) and categorical variables (presented as absolute numbers and percentages). We conducted Chi-square and Fisher's exact tests for categorical variables and Student's t-test for continuous variables. IBM SPSS for Windows statistical package, version 22.0, was used for data analysis (SPSS, Inc., Chicago, Illinois, United States). To test the hypothesis that a significant proportion of depressive symptoms are related to health factors in older adults with DM, the association between depressive symptoms and the possible risk factors among individuals with DM was assessed using Pearson's chi-square test in bivariate analysis. The independent variables with P < 0.2 in the bivariate analysis were entered into a binary logistic regression model using the stepwise regression method. The calculation of the odds ratio (OR) and statistically significant differences (P < 0.05) were considered in the absence of overlapping 95% confidence interval (CI) for all analyses.

### Ethical considerations

The study was approved by the Research Ethics Committee of the Ana Nery Hospital, under protocol number 1.953.841, on March 8, 2017, and adhered to the Helsinki guidelines at all times. All participants signed an informed consent form before participating in the study.

## RESULTS

The final sample comprised 236 older adults with DM. Most were female (76.7%). The mean age was 71.6 years (± 8.03). Of the sample, 64.0% declared brown ethnicity, 81.4% did not have a partner, and 61.9% received primary or lower education.

Depressive symptoms were reported in 24.2% of older adults with DM. Table 1 shows the characteristics of the study population according to depressive symptoms. Being female without a partner was predominant, although it was not significantly associated with depressive symptoms. Brown ethnicity among older adults was primarily associated with depressive symptoms.

**Table 1:** Distribution and association of sociodemographic characteristics of older adults with diabetes mellitus according to depressive symptoms

		Depressive symptoms		P value
		No [n (%)]	Yes [n (%)]	
**Sex**				
	Female	132 (73.7)	49 (86.0)	0.057
	Male	47 (26.3)	8 (14.0)	
**Ethnicity**				
	Brown	117 (34.0)	34 (59.6)	0.037*
	Black	34 (19.0)	9 (15.8)	
	White	28 (15.6)	11 (19.3)	
	Other	0 (0.0)	3 (5.3)	
**Marital status**				
	Without partner	144 (80.4)	48 (84.2)	0.525
	With partner	35 (19.6)	9 (15.8)	
**Education level**				
	≥ Elementary school	72 (40.2)	18 (31.6)	0.242
	≤ Primary education	107 (59.8)	39 (68.4)	

* P < 0.05.

Table 2 presents the behavioral characteristics of the study population. Only alcohol consumption was associated with depressive symptoms.

**Table 2:** Distribution and association of behavioral characteristics of older adults with diabetes mellitus according to depressive symptoms

		Depressive symptoms		P value
		No [n (%)]	Yes [n (%)]	
**Religion**				
	Catholic	70 (39.1)	18 (31.6)	0.076
	Protestant	80 (44.7)	22 (38.6)	
	Not practicing	29 (16.2)	17 (29.8)	
**Financial difficulty**				
	Yes	82 (45.8)	21 (36.8)	0.234
	No	97 (54.2)	36 (63.2)	
**Physical activity**				
	Yes	52 (29.1)	13 (22.8)	0.358
	No	127 (70.9)	44 (77.2)	
**Smoking**				
	Smoker	6 (3.4)	8 (8.8)	0.164
	Former smoker	68 (38.0)	24 (42.1)	
	Never smoked	105 (58.7)	28 (49.1)	
**Alcohol consumption**				
	Excessive	2 (1.1)	4 (7.0)	0.032*
	Moderate	13 (7.3)	2 (3.5)	
	Non-consumer	164 (91.6)	51 (89.5)	

* P < 0.05.

**Figure 1:**
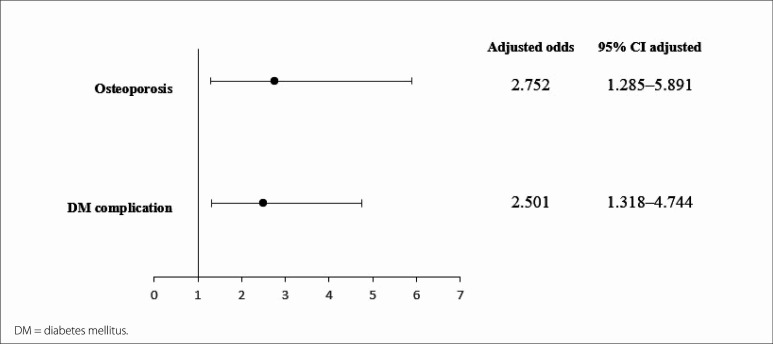
Odds ratio and 95% confidence interval (CI) of final regression model for risk of depressive symptoms.

Table 3 shows the characteristics of the population's health conditions. The existence of any diabetes complications and ocular and circulatory types of DM complications were significantly associated with depressive symptoms. Among comorbidities, rheumatism, osteoporosis, and heart and circulation problems were associated with depressive symptoms. Difficulty sleeping and severe chronic pain were predominant among those with depressive symptoms and were significantly associated with depressive symptoms. The final multivariate analysis model is presented in Figure 1, which shows the 95% confidence indices of each variable that remained in the model as well as the OR. Notably, the 95% CI coefficients were attenuated; however, DM complication along with osteoporosis remained associated with depressive symptoms.

**Table 3:** Distribution and association of health conditions of older adults with diabetes mellitus according to depressive symptoms

		Depressive symptoms		P value
		No [n (%)]	Yes [n (%)]	
**DM family history**				
	Yes	80 (59.8)	38 (66.7)	0.550
	No	11 (6.1)	4 (7.0)	
	Do not know	61 (34.1)	15 (26.3)	
**Treatment**				
	Oral	156 (77.2)	53 (79.1)	0.228
	Insulin	32 (15.8)	11 (16.4)	0.809
	Non-medicated	3 (1.5)	0 (0.0)	0.325
	None	11 (5.5)	3 (4,5)	0.806
**DM complication**				
	Yes	69 (38.5)	34 (59.6)	0.005*
	No	110 (61.5)	23 (40.4)	
**Complication type**				
	Renal	7 (7.5)	4 (7.4)	0.333
	Ocular	31 (33.0)	26 (48.1)	0.000*
	Circulatory	42 (44.7)	21 (38.9)	0.047*
	Diabetic foot	10 (10.6)	2 (3.7)	0.534
	Amputation	4 (4.2)	1 (1.9)	0.826
**Rheumatism**				
	Yes	50 (27.9)	29 (50.9)	0.001*
	No	129 (72.1)	28 (49.1)	
**Osteoporosis**				
	Yes	26 (14.5)	20 (35.1)	0.001*
	No	153 (85.5)	37 (64.9)	
**Hypertension**				
	Yes	146 (81.6)	49 (86.0)	0.445
	No	33 (18.4)	8 (14.0)	
**Circulation problems**				
	Yes	76 (42.5)	35 (61.4)	0.013*
	No	103 (57.5)	22 (38)	
**Heart problems**				
	Yes	33 (18.4)	19 (33.3)	0.018*
	No	146 (81.6)	38 (66.7)	
**Difficulty sleeping**				
	Yes	82 (45.8)	37 (64.9)	0.012*
	No	97 (54.2)	20 (35.1)	
**Vision problems**				
	Yes	80 (44.7)	32 (56.1)	0.132
	No	99 (55.3)	25 (43.9)	
**Chronic pain**				
	Yes	81 (45.3)	44 (77.2)	0.000*
	No	98 (54.7)	13 (22.8)	

* P < 0.05; DM = diabetes mellitus.

## DISCUSSION

This study identified a 24.2% prevalence of depressive symptoms in older adults with diabetes and demonstrated a significant association between DM complications and osteoporosis as a health comorbidity.

Studies conducted among older adults in Brazil have shown a prevalence of depressive symptoms ranging from 13% to 39% among community-dwelling older adults. In the present study, the prevalence of depressive symptoms among older adults with DM was 24.2%, which is within the Brazilian average range. Studies reported a 30% and 34.4% prevalence of depressive symptoms in older adults enrolled in the Hiperdia program^22^ and those assisted by the Family Health Strategy, respectively.^24^ Both studies were conducted in primary care and used the GDS-15 to investigate the prevalence of depressive symptoms. This shows that the prevalence rates of depressive symptoms among older adults with DM are significantly higher than in those without any chronic disease. Importantly, this can lead to debilitating conditions because of poor metabolic control and the emergence of other health complications resulting from the absence or decrease of treatment adherence, decreased social bonds, and inadequate diet. These negative outcomes have been consistently observed in the relationship between depressive symptoms and poorer self-care among older adults with diabetes, and could be explained by difficulties in maintaining proactive and effective self-care behaviors.^25,26^ In the present study, older adults with DM complications were more susceptible to developing depressive symptoms than those without complications. Diabetes complications and depression are reportedly a bi-directional relationship, and the risk of depression is higher in people with diabetes complications, and vice versa.^27^ Meta-analysis studies indicate that diabetes increases the risk of developing depression by approximately 25%.^28,29^ Moreover, the risk of complications is higher when both diabetes and depression are present. Individuals with DM have a 36% higher risk of developing microvascular complications, such as nephropathy, retinopathy, and neuropathy. Researchers observed a 25% increase in the risk of developing macrovascular complications, such as peripheral vascular disease, erectile dysfunction, and coronary artery disease.^30–32^ As noted, there is strong evidence that these comorbidities are linked with disability and loss of years of life.^33^ Notably, people with diabetes and symptoms of depression have higher levels of diastolic blood pressure, triglycerides, glycated hemoglobin, higher body mass index, and worse glycemic control. Therefore, older adults are considered at risk for DM complications and other comorbidities that can significantly compromise their health and quality of life.^19,20^ Moreover, depressive symptoms may appear even before the diagnosis of DM or during the onset of complications, depending on the individual or the course of the disease.^34,35^

Among the health comorbidities evaluated in this study, osteoporosis remained in the final model even after adjustment, showing an increased risk for depressive symptoms in older adults with DM. This comorbidity is predominantly cited by older adults in aging studies,^7,36^ including being associated with diabetes itself.^37,38^

The presence of osteoporosis combined with connective tissue problems, neuropathies, and vasculopathies may increase the incidence of complications in older adults with diabetes. This further contributes to their limitations and restricted autonomy, functional disability, fragility, and the potential development of depressive symptoms.^39,40^

Osteoporosis commonly causes pain, which directly affects the quality of life of older adults with diabetes. Furthermore, complementary data in this study showed that 77.2% of older adults with depressive symptoms had self-reported chronic pain. Whether this pain is linked to musculoskeletal pain or complications of DM, it remains a primary reason for older adults to seek health services.^37,41,42^ Thus, this study expands the knowledge that the presence of osteoporosis and diabetes complications in older adults can be associated with depressive symptoms. Moreover, when older adults seek health services, health professionals must critically examine these associations and employ a holistic approach, for example, by testing for depressive symptoms.

In this context, testing for depressive symptoms in individuals with diabetes to enable early detection and treatment is one of the challenges faced by primary healthcare professionals. Lack of screening may be attributed to absent or limited training in mental health issues, inability or lack of skills to use mental health assessments, and difficulties in distinguishing depression symptoms or diabetes complications from symptoms of physical illness. Ideally, patients with diabetes should be referred to mental health consultations and supported in self-management education, which can provide them with an increased ability to maintain their treatments and identify coping strategies for depressive symptoms.^43,44^

## CONCLUSION

The present study findings are broadly consistent with data from national and international literature, showing a significant prevalence of depressive symptoms in older adults with type 2 DM. In conclusion, this study provides strong evidence that complications of DM significantly increase the risk of depressive symptoms in older adults, especially those with DM and osteoporosis. This perspective suggests that, by identifying groups at greater risk, primary care professionals can develop care strategies and refer older adults with DM for a mental health consultation to reduce complications and improve prognosis. In the present study, individuals with DM at a higher risk for the development of depressive symptoms were represented among those with complications arising from DM and musculoskeletal comorbidities, such as osteoporosis.
